# Electrochemical Based Biosensors

**DOI:** 10.3390/bios2030269

**Published:** 2012-07-24

**Authors:** Chung Chiun Liu

**Affiliations:** Department of Chemical Engineering and Electronics Design Center, Case Western Reserve University, Cleveland, OH 44106, USA; E-Mail: cxl9@case.edu; Tel.: +1-216-368-2935

**Keywords:** electrochemical biosensor, microfabrication, nano-catalyst, antibody, antigen

## Abstract

This editorial summarizes the general approaches of the electrochemical based biosensors described in the manuscripts published in this Special Issue. Electrochemical based biosensors are scientifically and economically important for the detection and early diagnosis of many diseases, and they will be increasingusedand developed in the coming years. The importance of the selection of recognition processes, fabrication techniques and biosensor materials will be introduced.

## 1. Introduction

This Special Issue has received and published manuscripts relating to electrochemical based biosensors [1,2,3,4,5,6]. The recognition process or the reaction mechanism that these biosensors employ include ZnO based enzymatic reaction, carbon nano-tube enzymatic-gelatin reaction and whole cell system. The fabrication techniques used in these developments include micro-fabrication and printing technologies. Nano-tube and nano-particles are incorporated in these advancements. This Special Issue intends to bring the importance of electrochemical based sensors, which are the critical parts of many successful biosensors, to the attention of biosensor researchers. Electrochemical based sensors provide the detection mechanism for the biosensor. This detection mechanism can be incorporated by a recognition process with the analyte through an enzymatic reaction, antibody and antigen interaction, whole cell recognition process or otherwise, as illustrated by the selected manuscripts. A very successful example of an electrochemical based biosensor is the blood glucose strip (single-use, disposable biosensor) currently used for diabetic patient management.In this biosensor, the enzymatic reaction with blood glucose generates H_2_O_2_, which is then electrochemically oxidized producing a current to quantify the blood glucose.

In the past years, the identification of various biomarkers for different diseases has opened up opportunities for the use of biosensors to detect these diseases, and the recognition mechanism of electrochemical based techniques cannot be overlooked. The thick- and thin-film metallization processes, the silicon based micro-fabrication and MEMS technologies contribute to the construction of different electrochemical based biosensors. The selection of sensor construction materials and the choice of suitable nano-particle catalysts also lead to enhanced feasibility to detect biomarkers by electrochemical means. The advancements of electronics allow the miniaturization of the biosensors to become a reality. 

Therefore, we believe that electrochemical based biosensors will be important and useful for the scientific and economical advancements of biosensors in the future years together with contributions from other technologies. In this brief editorial, we wish to highlight the essential characteristics of an electrochemical based biosensor to assist researchers in developing and advancing suitable biosensors for unique applications.

## 2. Electrochemical Sensing Techniques, and Sensor Fabrication

Coulometric, potentiometric and amperometric sensing mechanisms can be used as electrochemical based biosensors. Researchers have a choice of any of these electrochemical sensing mechanisms to meet their specific needs based on the sensitivity, selectivity and the cost of the biosensor required.For instance, potentiometric measurements can be simply usedand the sensor is relatively simple to fabricate. However, this method has a limited sensitivity (semi-logarithm relationship between sensor output and activity of analyte). On the other hand, amperometric measurement can yield a linear relationship between the output of the sensor and the activity (concentration) of the analyte, but the surface area of the sensing electrode element must be well controlledto allow reproducible results. Therefore, researchers must choose the appropriate sensing method to meet their needs prior to design and construction of the electrochemical based biosensor. 

Electrochemical based biosensors can have a two- or three-electrode configuration. In the two-electrode arrangement, the working and the reference (counter) electrodes are used, whereas ina three-electrode configuration, working, counter and reference electrodes are used. As expected, the two electrode system can be smaller, but the stability of the applied potential may suffer. Again, researchers need to assess the needs, the space available, and the importance of the stability of the applied potential. These considerations will aid in the biosensor design and the selection of the mode of operation. 

## 3. Fabrication of Electrochemical Based Biosensors

Electrochemical based biosensors often contain a metal or metallic oxide detecting (working) electrode element with or without nano-particle metallic catalysts or immobilized enzyme, antibody, antigen and whole cell. Both the thick-film and thin-film fabrication processes allow cost-effective production of the biosensor. The needs and demand of the biosensor will govern the choice of the fabrication technique. For instance, thick-film screen printing or ink-jet printing processesare relatively inexpensive and suitable for a single use, disposable biosensor such as the blood glucose biosensor. However, thick-film printing has an inherent variation concern due to the binder material used; therefore, researchers need to assess whether this inherent variation will affect the sensitivity of the biosensor output. On the other hand, the thin-film process employs atomic or molecular deposition, which is more reproducible yet more costly to process. Therefore, researchers must first determine the goals of the detection, the sensitivity required, the quantity, and the amount that can be spent onthe biosensors. 

## 4. Selection of the Biosensor Component Materials and Catalysts

Regardless of whether the electrochemical based biosensor uses a two- or three-electrode configuration, appropriate materials must be chosen for the construction of the electrode elements.For this selection, the mode of detection method and the fabrication process must be taken into consideration, in addition to the needs of the biosensor. Hence, the selection of the materials for the components of the electrochemical based biosensor requires consideration beforethe development of the biosensor. The choice of the materials for the working and the reference electrode is important and will directly affect the biosensor measurement and output. For any *in vivo* applications, the biocompatibility is an additional consideration.If the biosensor requires a metallic catalyst to enhance its performance, the size and composition of the metallic catalyst as well as its adaptation to the fabrication process will need to be evaluated. If a biological catalyst, such as enzyme, antibody, whole cell *etc.*, is used, the immobilization process will need to be considered.

It is generally acknowledged that a large sensing surface area of the detecting sensing element (working electrode) is desirable. Consequently, in addition to nano-particle catalysts, carbon nano-tubes are often used for this purpose. However, the reproducibility of the carbon nano-tube and the fabrication technique employed in this development are important to ensure that the biosensors can be manufactured uniformly.

The advancement of an electrochemical based biosensor requires much consideration. We have pointed out that the mode of the recognition process, the fabrication technology, and the materials chosen for the sensing elements should first be assessed carefully before any further development. We believe that electrochemical sensing is a critical part of biosensor research, and the electrochemical based biosensors will play an important role in biosensor development in the years to come.

## References

[1] Ibupoto Z.H., Ali S.M.U., Khun K., Chey C.O., Nur O., Willander M. (2011). ZnO nanorods based enzymatic biosensor for selective determination of penicillin. Biosensors.

[2] Oliveira J.E., Mattoso L.H.C., Medeiros E.S., Zucolotto V. (2012). Poly(lactic acid)/carbon nanotube fibers as novel platforms for glucose biosensors. Biosensors.

[3] De Wael K., De Belder S., Pilehvar S., van Steenberge G., Herrebout W., Heering H.A. (2012). Enzyme-gelatin electrochemical biosensors: Scaling down. Biosensors.

[4] Wang J., Wu C., Hu N., Zhou J., Du L., Wang P. (2012). Microfabricatedelectrochemical cell-based biosensors for analysis of living cells *in vitro*. Biosensors.

[5] Yarman A., Neumann B., Bosserdt M., Gajovic-Eichelmann N., Scheller F.W. (2012). Peroxide-dependent analyte conversion by the Hemeprosthetic group, the Hemepeptide “microperoxidase-11” and Cytochrome c on Chitosan capped gold nanoparticles modified electrodes. Biosensors.

[6] Stein N.E., Hamelers H.V.M., van Straten G., Keesman K.J. (2012). Effect of toxic components on microbial fuel cell-polarization curves and estimation of the type of toxic inhibition. Biosensors.

